# Tumour–stroma interactions in colorectal cancer: converging on *β*-catenin activation and cancer stemness

**DOI:** 10.1038/sj.bjc.6604401

**Published:** 2008-05-27

**Authors:** N H Le, P Franken, R Fodde

**Affiliations:** 1 Department of Pathology, Josephine Nefkens Institute, Erasmus Medical Centre, Erasmus MC, Rotterdam, The Netherlands

**Keywords:** colorectal cancer, *β*-catenin, cancer stem cells, stroma, fibroblast

## Abstract

Sporadic cases of colorectal cancer are primarily initiated by gene mutations in members of the canonical Wnt pathway, ultimately resulting in *β*-catenin stabilisation. Nevertheless, cells displaying nuclear *β*-catenin accumulation are nonrandomly distributed throughout the tumour mass and preferentially localise along the invasive front where parenchymal cells are in direct contact with the stromal microenvironment. Here, we discuss the putative role played by stromal cell types in regulating *β*-catenin intracellular accumulation in a paracrine fashion. As such, the tumour microenvironment is likely to maintain the cancer stem cell phenotype in a subset of cells, thus mediating invasion and metastasis.

In the intestinal tissue architecture, epithelial cells lining the luminal surface are tightly regulated to ensure homeostasis. Epithelial cell renewal is fuelled by an adult stem cell compartment localised at the bottom of the crypt (Potten and Loeffler, 1990; Barker *et al*, 2007). As cells migrate upwards, after a transient proliferative phase, the epithelium differentiates into specialised cell types including absorptive enterocytes, mucus secreting goblet cells, and enteroendocrine cells. This migratory process with concomitant differentiation is finalised when cells reach the top of the crypt where they are exfoliated into the lumen upon apoptosis. In the upper gastrointestinal tract, Paneth cells represent an exception as they move downwards while differentiating. Overall, cell renewal, proliferation, and differentiation are coupled to positional localisation along the crypt-to-villus axis. In fact, this positional regulation of proliferation and differentiation can be correlated to gradients in the degree of activity of several signalling pathways known to govern stemness and differentiation, including Wnt/*β*-catenin, bone morphogenic protein (BMP), Notch, and transforming growth factor-*β* (TGF*β*; Crosnier *et al*, 2006). The mesenchyme plays a complex role in the positional gradient of signalling ligand availability. Intestinal subepithelial myofibroblasts are specialised stromal cells that form a continuous sheet directly localised underneath the mucosa. These myofibroblasts contribute to epithelial cell function by providing mechanical support and secreting key signalling ligands. Thus, the intimate interaction between the parenchyme and mesenchyme ensures proper tissue function, balancing cell renewal, and differentiation. Activation of canonical Wnt signalling characterises the base of the intestinal crypt as shown by the nuclear *β*-catenin localisation crypt cells (Figure 1). Note that Paneth cells in the small intestine also show nuclear *β*-catenin accumulation as previously reported (Van Es *et al*, 2005). Moving upwards along the crypt-to-villus axis, terminal differentiation coincides with the more restricted membrane-bound *β*-catenin localisation and its absence in the nucleus and cytosol (Figure 1). Recently, it has been reported that the BMP antagonists, gremlin 1, gremlin 2, and chrodin-like 1, are selectively expressed by crypt-based myofibroblasts and smooth muscle cells (Kosinski *et al*, 2007). Moreover, Gremlin 1 was shown to activate Wnt/*β*-catenin signalling in normal rat intestinal epithelial cells, thus indicating that stromal-derived factors regulate Wnt/*β*-catenin-signalling activity in the intestinal stem cell niche.

Upon constitutive activation of the Wnt signalling route, intestinal homeostasis is disturbed, paving the way for pathogenesis. Indeed, the vast majority of sporadic colorectal cancer cases is caused by constitutive Wnt activation due to mutations in either the *APC* tumour suppressor or the *β*-catenin (*CTNNB1*) oncogene (Fodde *et al*, 2001). Loss of *APC* function leads to destabilisation of the ‘destruction complex’, a multiprotein complex encompassing three scaffold proteins, APC, Axin1, and Axin2 (conductin), and two kinases, glycogen synthase kinase-3*β* (GSK3*β*) and casein kinase 1 (CK1). The complex binds and phosphorylates *β*-catenin at serine and threonine residues, thus targeting it for ubiquitination and proteolytic degradation. In contrast, oncogenic mutations in *β*-catenin render it resistant to Ser/Thr phosphorylation and proteolytic degradation. Upon its cytoplasmic stabilisation and subsequent nuclear translocation, *β*-catenin binds to members of the TCF/LEF family of transcription factors, thus modulating expression of a broad range of target genes (http://www.stanford.edu/~rnusse/pathways/targets.html). Although the presence of these initiating mutations predicts nuclear *β*-catenin accumulation throughout the tumour mass, heterogeneous intracellular distributions are observed within primary colorectal tumours and their metastases. In particular, tumour cells located at the invasive front and those migrating into the adjacent stromal tissue are earmarked by nuclear *β*-catenin accumulation (Brabletz *et al*, 1998, 2001; Figure 1). Hence, different levels of Wnt/*β*-catenin-signalling activity are likely to reflect tumour heterogeneity and to underlie malignant behaviour (Gaspar and Fodde 2004; Brabletz *et al*, 2005a).

Several intrinsic (cell autonomous and/or autocrine) and extrinsic (paracrine, derived from the tumour microenvironment) factors may explain the observed heterogeneity of Wnt/*β*-catenin-signalling activity within the tumour mass (Fodde and Brabletz, 2007). Here, we discuss stromal factors likely to play a role in the heterogeneous *β*-catenin intracellular localisation and signalling activity in tumour cells. As such, the tumour microenvironment may drive tumour growth and even selectively support a subset of tumour cells, the cancer stem cells (CSCs), thus actively contributing to malignancy.

## STROMAL CELLS AFFECTING TUMOUR GROWTH, NUCLEAR *β*-CATENIN ACCUMULATION, AND CANCER STEMNESS

As stated above, stromal regulation significantly contributes to the preservation of normal tissue architecture. Myofibroblasts, for example, are not only tightly associated with the intestinal epithelium thus ensuring homeostasis through reciprocal interactions, but are also essential for wound healing upon tissue injury, when they are transiently enriched and activated (Gabbiani, 2003). Expression of *α*-smooth muscle actin (*α*-SMA) characterises these myofibroblasts and underlies contractile force tension that facilitates healing. Myofibroblasts produce a variety of growth factors, prostaglandins, cytokines, chemokines, and extracellular matrix components that facilitate tissue repair and survival. Myofibroblasts arise through a multitude of processes, including transdifferentiation of resident fibroblasts, epithelial-to-mesenchymal transition (EMT) of parenchymal cells, recruitment, and differentiation of pericytes (progenitor cells localised at vascular sinuses), and from bone marrow-derived circulating immature fibrocytes (Desmouliere *et al*, 2004). Upon completion of the wound healing process, myofibroblasts revert back to their dormant state. In fact, tumorigenesis has been described as a condition comparable to an open wound of chronic nature (Dvorak, 1986). Accordingly, fibroblasts are one of the most abundant cell types in the stromal microenvironment associated with solid tumours (Adegboyega *et al*, 2002; De Wever and Mareel, 2003; Kalluri and Zeisberg, 2006). In response to the malignant lesion within the epithelial compartment, stromal fibroblasts become morphologically ‘activated’. Similar to the wound-healing process, an activated response of the tumour stroma may initially be triggered in an attempt to restore tissue homeostasis. However, as the tumour progresses, the microenvironment is more likely to become a ‘partner in crime’ in malignancy. A subset of tumour stromal fibroblasts, also referred to as cancer-associated fibroblasts (CAFs), peritumoral fibroblasts, reactive stromal fibroblasts, tumour-associated fibroblasts, or myofibroblasts, acquire distinct phenotypic characteristics. These cells share many of the properties of normal myofibroblasts such as *α*-SMA expression and increased production of growth factors, and of a variety of matrix remodelling proteases, which facilitate migration and invasion of the tumour cells (De Wever and Mareel, 2003; Desmouliere *et al*, 2004; Mukaratirwa *et al*, 2005). In view of their specific growth promoting effects, CAFs are primary candidates for locally modulating Wnt/*β*-catenin signalling, resulting in heterogeneous patterns of *β*-catenin intracellular localisation within colorectal tumours (Brabletz *et al*, 2001). Convergence of CAFs in specific regions of the tumour may provide a local increase in ligand availability that directly, in the case of Wnt ligands, or indirectly, in the case of growth factors, prostaglandins, and chemokines, may cross talk with and increase Wnt/*β*-catenin signalling.

Cross talk of a variety of factors has been reported to modulate nuclear *β*-catenin accumulation. For instance, hepatocyte growth factor or scatter factor (HGF, SF) induces *β*-catenin stabilisation in colorectal cancer cells via c-MET-dependent inhibition of GSK*β* activity and its Tyr phosphorylation (Rasola *et al*, 2007). Tyr phosphorylation of *β*-catenin leads to its stabilisation and nuclear signalling activity by decreasing its binding affinity to E-cadherin and the APC/GSK*β*/Axin destruction complex (Coluccia *et al*, 2007). Platelet-derived growth factor (PDGF) stimulation of HT-29 colorectal cancer cells increases *β*-catenin activation via p68-dependent inhibition of Ser/Thr phosphorylation by GSK3*β* (Yang *et al*, 2006).

In addition to the secretion of growth factors capable of modulating *β*-catenin stabilisation during tumour growth and local invasion, CAFs may also play a significant role in the metastatic process. As stated above, these cells can originate from circulating precursor cells recruited from the bone marrow, often referred to as fibrocytes. Therefore, these mesenchymal cells may not only exert local effects within the tumour, but could also represent systemic effectors relevant for the metastatic process by functioning as carrier cells during extravasation of tumour cells and/or ‘landscaping’ secondary organ sites where circulating tumour cells can home to and form secondary outgrowths. This may be of particular importance in view of the CSC hypothesis, which predicts that only a subset of tumour cells, displaying stem cell characteristics, will be successful in invading surrounding tissues and forming metastases in secondary organs. We have previously postulated that cancer stemness may be conferred by specific levels of *β*-catenin activation in colorectal cancer (Brabletz *et al*, 2005a; Fodde and Brabletz, 2007). Stromal cells may play a significant role by providing a supportive microenvironment that maintains CSCs at the primary tumour site and also underlies their invasive behaviour and spreading to distant sites. Karnoub *et al* (2007) have recently shown that bone marrow-derived mesenchymal stem cells can indeed increase metastatic potency of breast tumour cells. In addition, Kaplan *et al* (2005) have reported that haematopoietic progenitor cells expressing vascular endothelial growth factor receptor-1 are recruited and home to premetastatic niches prior to the arrival of tumour cells in mice injected with Lewis lung carcinoma or B16 melanoma cells. This response directs the metastatic pattern and is triggered by tumour-specific secreted factors. These data indicate that stromal (precursor) cells are active coconspirators in malignancy by increasing metastatic potential of tumour cells and providing a ‘congenial soil’ for secondary growth.

As stromal cells may significantly modulate both tumour growth and nuclear *β*-catenin accumulation and thus represent a cancer stemness determinant, specific stromal cell characteristics may be selected during tumorigenesis to provide a supportive microenvironment for pathogenic events. For instance, selective pressure from the tumour promotes genetic loss of *p53* in stromal fibroblasts giving rise to highly proliferative stromal compartments in a mouse model for prostate cancer (Hill *et al*, 2005). Transforming growth factor-*β*is also highly expressed in most solid tumours and is capable of transforming fibroblasts towards an activated phenotype (De Wever and Mareel, 2003; Mishra *et al*, 2005; Orimo and Weinberg, 2006). Accordingly, stromal expression of the TGF-*β* type II receptor (TGFRII) reflects its activation by TGF*β* stimulation and directly correlates with prognosis and survival in human colorectal cancer (Bacman *et al*, 2007). Stromal abrogation of TGFRII leads to prostate and stomach tumours in a murine model (Bhowmick *et al*, 2004). Moreover, expression of PDGFR (platelet-derived growth factor receptor) in stromal cells directly correlates with advanced stage disease in human colorectal cancer (Kitadai *et al*, 2006a). Both a DNA vaccine against PDGFR*β* (Kaplan *et al*, 2006) as well as PDGFR inhibition by imatinib alone or in combination with irinotecan (Kitadai *et al*, 2006b), suppressed growth and dissemination of human colorectal cancer cells injected into mice, suggesting that increased PDGF signalling to stromal cells is a determinant for malignancy. Therefore, reciprocal interactions between tumour cells and the microenvironment facilitate tumour growth, invasion, and metastasis, by selecting not only for tumour cells capable of invasion and metastasis, but also for a stromal cell compartment that optimally supports the malignant phenotype.

In line with the above, EMT drives tumour cells towards a more mesenchymal phenotype and is implicated in invasive and malignant behaviour. It has been shown that colorectal cancer cells with nuclear *β*-catenin accumulation clustered along the invasive front undergo EMT as they detach from the tumour mass and invade the surrounding stroma (Brabletz *et al*, 2005b). Moreover, hepatocytes that have undergone TGF*β*-induced EMT and have acquired a fibroblastoid phenotype, show nuclear *β*-catenin accumulation, proliferation, and migration upon PDGF treatment (Fischer *et al*, 2007). S100A4, a mesenchymal gene expressed during EMT and associated with poor prognosis in colorectal cancer, is in itself a target gene of Wnt/*β*-catenin signalling (Stein *et al*, 2006). Therefore, EMT may determine a ‘double jeopardy’ effect: CSCs earmarked by nuclear *β*-catenin accumulation can transdifferentiate, thus generating a permissive niche capable of eliciting nuclear *β*-catenin translocation in other parenchymal cells located in direct contact with the stromal tumour microenvironment.

## IMMUNE CELLS AND ADIPOCYTES MODULATING TUMOUR GROWTH

Besides stromal fibroblasts, the tumour microenvironment consists of a variety of cell types capable of modulating tumour growth and possibly cancer stemness. Tumour-infiltrating innate and adoptive immune cells may confer both tumour-growth-promoting and -inhibiting effects (de Visser *et al*, 2006). Intuitively, increased infiltration of T cells should correlate with improved tumour clearance and prognosis, as recently reported in colorectal cancer cohorts (Pages *et al*, 2005; Clarke *et al*, 2006; Galon *et al*, 2006). However, a subtype of T cells, termed regulatory T cells (Tregs), are likely to exert tumour-growth-promoting effects due to their immune suppressive function to mediate self-tolerance, prevent autoimmunity, and enable the presence of a commensal bacterial flora in the intestine (Powrie, 2004). Regulatory T cells have been reported to be increased in peripheral blood and infiltrating lymphocytes among colorectal cancer patients (Ling *et al*, 2007). The increased presence of these Tregs sets the stage for immune evasion by tumour cells.

Several other tumour-infiltrating immune cells have been reported to support tumour growth, including tumour-associated macrophages, and immature myeloid and dendritic cells (Lizee *et al*, 2006). Also, it has been reported that specific functional aspects of innate immune cells are pivotal for intestinal homeostasis, inflammation, and tumorigenesis. Exemplary, a T-cell-specific knockout mouse model of *Smad4*, a downstream component of TGF*β* and BMP signalling, resulted in intestinal tumorigenesis (Kim *et al*, 2006). Recently, Kitamura *et al* (2007) showed that immature myeloid cells (iMCs) are recruited from the bone marrow to the tumour invasion front of compound heterozygous *cis*-*Apc*^+/Δ*716*^*;Smad4*^+/−^ mice with invasive intestinal adenomacarcinoma. These CD34^+^ iMCs promote tumour growth by expression of the matrix metalloproteinases, MMP9 and MMP2, and the CC-chemokine receptor 1 (CCR1), and migrate towards the CCR1 ligand CCL9, highly increased in the tumour epithelium. Greten *et al* (2004) reported that activation of the transcription factor NF*κ*B (nuclear factor-*κ*B), a key mediator of inflammation, has a critical role in the development of tumours resulting from chronic inflammation or exogenous mutagens, induced by exposure to dextran sulphate sodium salt and Azoxymethane (AOM). Moreover, genetic ablation of MyD88, a signalling adaptor of Toll-like receptors in the innate immune system, was shown to reduce mortality due to intestinal tumorigenesis in *Apc*^+/*Min*^ mice (Rakoff-Nahoum and Medzhitov, 2007). In fact, Auguste *et al* (2007) have shown that liver metastases formation coincides with an inflammatory, TNF*α*-mediated, host organ response. This inflammatory reaction upregulates cell adhesion molecules in the liver stromal microvasculature and supports tumour cell arrest and extravasation in a metastatic mouse model induced by intrasplenic injection of the highly metastatic human colorectal cancer cell line, CX-1. Finally, adipocytes have also been reported to promote proliferation of colon cancer cells (Amemori *et al*, 2007).

Overall, these data indicate that the host inflammatory response is a key mediator of tumour survival, extravasation, and metastasis formation. In fact, a broad spectrum of diverse cell types from within the tumour microenvironment may contribute to the modulation of *β*-catenin activation and cancer stemness, thus promoting intestinal tumour progression and even initiation. In this regard, tumour progression and cancer stemness may significantly be determined by a context-dependent modulation of *β*-catenin activity in colorectal cancer.

## HYPOXIA- AND OXIDATIVE STRESS-INDUCED SELECTION OF TUMOUR CELLS DISPLAYING NUCLEAR *β*-CATENIN ACCUMULATION AND CANCER STEMNESS

Besides the specific stromal cell types, other factors from within the tumour microenvironment are likely to play significant roles in promoting cancer stemness and malignant behaviour through *β*-catenin nuclear accumulation and signalling. Hypoxia underlies progressive tumour growth in the majority of solid tumours (Sullivan and Graham, 2007). During tumour growth, certain areas are exposed to reduced oxygen tension due to a disturbed microcirculation and inadequate blood supply. Hypoxic conditions are often found in the invasive front of colorectal carcinomas in association with stabilisation of the HIF1*α* (hypoxia-inducible factor-1*α*) transcription factor (Sivridis *et al*, 2005). HIF1*α* stabilisation results in transcriptional regulation of a variety of target genes, including the proangiogenic factors vascular endothelial growth factor and PDGF (Koukourakis *et al*, 2006). In fact, Cleven *et al* (2007), showed that expression of HIF1*α* in the stromal compartment correlates with poor prognosis in colorectal cancer. Moreover, loss of MUTYH function, a DNA glycogylase involved in base excision repair caused by oxidative stress, results in increased susceptibility to spontaneous and oxidative stress-induced (by the oxidative reagent KbrO3) intestinal tumorigenesis (Sakamoto *et al*, 2007). These data indicate that hypoxia and oxidative stress play a pivotal role in colorectal cancer progression. Notably, Kaidi *et al* (2007) reported that HIF1*α* binds directly to *β*-catenin in the nucleus, thus linking hypoxia-induced cellular changes to *β*-catenin activation.

## BIOLOGICAL EFFECTS OF NUCLEAR *β*-CATENIN ACCUMULATION

The nuclear accumulation of *β*-catenin observed in colorectal cancer cells distributed along the invasive front may not only reflect a specific level of canonical Wnt activity but also indicate the activation of additional signalling pathways. It has been shown that, in the nucleus, *β*-catenin binds to a broad spectrum of transcription factors other than TCF and LEF and modulates a plethora of downstream targets possibly contributing to cancer stemness and malignancy (Table 1). As stated above, hypoxia induces stabilisation of HIF1*α* and its interaction with *β*-catenin, thereby competing with TCF/LEF1 transcription factors for *β*-catenin binding in colorectal cancer cells (Kaidi *et al*, 2007). Stabilisation and binding of HIF1*α* to *β*-catenin results in inhibition of Wnt reporter activity, induction of cell-cycle arrest, survival, and cellular adaptation, and is likely to contribute to the malignant and invasive behaviour of tumour cells exposed to reduced oxygen tension. Similarly, oxidative stress stimulates *β*-catenin binding to the Forkhead box O transcription factors, inducing cell-cycle arrest and survival (Essers *et al*, 2005).

Through interaction with Smads (including Smad1, Smad3, and Smad4), *β*-catenin may also coregulate a subset of common TGF*β*, BMP, and Wnt target genes (Nishita *et al*, 2000; Hussein *et al*, 2003; Chakladar *et al*, 2005; Hu and Rosenblum, 2005). Transforming growth factor-*β* and BMP signalling are known to be important regulators of epithelial cell function. Synergism among TGF*β*, BMP, and Wnt signalling pathways may represent a significant determinant of malignant behaviour in tumour cells. *β*-Catenin binding to Smad7, an inhibitory molecule induced upon TGF*β* pathway activation as part of a negative feedback loop, has also been reported to be rate limiting for TGF*β*-induced apoptosis (Edlund *et al*, 2005) and induces proteolytic degradation of *β*-catenin (Han *et al*, 2006). When c-Jun, a stress- and growth factor-induced transcription factor, is recruited to the TCF/LEF1/*β*-catenin complex, synergistic effects on intestinal tumorigenesis are observed (Nateri *et al*, 2005). Also, gut-specific deletion of c-Jun decreased tumour multiplicity and increased life span in the *Apc*^*Min*^ mouse model for intestinal cancer. Recently, both c-Jun and its known heterodimerisation partner, c-Fos, were reported to bind directly to *β*-catenin (Toualbi *et al*, 2007). Therefore, binding of *β*-catenin to different interaction partners in the nucleus may direct both TCF/LEF1-dependent and -independent transcriptional regulation.

Hence, in view of this observed promiscuity for nuclear transcription factors (Table 1), *β*-catenin is likely to represent a central node where different signals converge and are subsequently coordinated to regulate tissue homeostasis under physiological conditions and cancer stemness in the context of tumour–stroma interactions. Because the putative *β*-catenin interaction partners are themselves regulated by extracellular stimuli, it is plausible that the subsequent effects on *β*-catenin activation and possibly cancer stemness are modulated in a context-dependent manner. In fact, *β*-catenin has been reported to interact directly with several growth factor receptors, including EGFR (epidermal growth factor receptor, ErbB1), Met (the receptor for HGF), TGFRII (the receptor that is activated upon TGF stimulation), and KIT (the receptor for stem cell factor; Hoschuetzky *et al*, 1994; Monga *et al*, 2002; Tian and Phillips, 2002; Kajiguchi *et al*, 2008). These interactions result in *β*-catenin Tyr phosphorylation, stabilisation, and increased transcriptional activity.

## CONCLUSIONS

Despite the clear genetic prerequisite for mutations in downstream components of the Wnt/*β*-catenin-signalling pathway that result in its constitutive activation, heterogeneous intratumour expression and subcellular localisation of *β*-catenin is commonly observed in colorectal cancer. Tumour cells located at the invasive front display increased nuclear *β*-catenin accumulation, suggesting that this nonrandom intracellular distribution earmarks and underlies tumour heterogeneity and malignancy. Therefore, it has been postulated that *β*-catenin may play a significant role in cancer stemness, driving invasion and metastasis. *β*-Catenin regulation is already known to be important during homeostasis, as Wnt/*β*-catenin signalling governs several adult stem cell niches, including the intestinal crypt. The tumour microenvironment may play a central role in the malignant transformation of tumour cells by locally modifying *β*-catenin activity at the primary tumour site as well as preparing secondary organ sites for metastatic growth. Individual ‘stromal signatures’, that is, characteristic of stromal cell function, inflammation, and other stress-induced responses, may determine disease progression, responsiveness to different (adjuvant) therapeutic strategies, and, thus, prognosis and survival for colorectal cancer patients. Stromal cells have even been suggested as possible targets for tailor-made therapeutic interventions for intestinal tumorigenesis, rather than parenchymal cells. Here, we propose that the functional characterisation of additional *β*-catenin-binding partners in these alleged CSCs will improve our understanding of malignancy and invasion and open future perspectives for a metastasis-free survival to colorectal cancer patients.

## Figures and Tables

**Figure 1:**
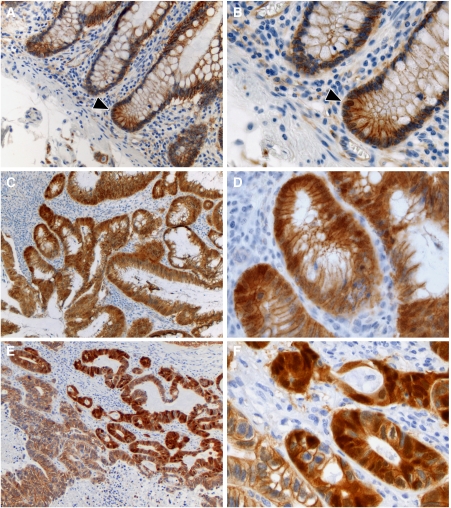
*β*-Catenin immunohistochemical staining of formalin-fixed, paraffin-embedded normal human colonic epithelia (panels **A** and **B**), and of a primary colorectal tumour (panels **C** and **D**) and a liver metastasis (panels **E** and **F**). The right panels contain magnifications (× 40) of specific areas from the left panels (× 20). The arrowheads in panels **A** and **B** indicate an epithelial cell localised at the base of the crypt with nuclear *β*-catenin accumulation. Both the primary colorectal tumour and liver metastasis (panels **C**–**F**) show nuclear *β*-catenin accumulation in cells invading the surrounding stroma, whereas other tumour cells display only membranous localisation.

**Table 1:** Nuclear *β*-catenin-binding partners

**Protein**	**Interaction and biological significance**	**Reference**
14-3-3-z	Binds *β*-catenin and stabilises it, Akt dependent	Tian *et al* (2004)
Akt	Phosphorylates *β*-catenin at S552 and increases cytoplasmic pool, increases binding to 14-3-3, increases transcriptional activity	Fang *et al* (2007)
AR (androgen receptor)	Binds *β*-catenin, augments AR activity, inhibits TCF-dependent transcription	Yang *et al* (2002)
AP1 and Smad3/4	Complex with *β*-catenin and TCF/Lef1 to activate gastrin target gene	Chakladar *et al* (2005)
BCL9 (Legless)	Binds *β*-catenin and TCF/LEF1, increases transcription, Pygopus dependent	Kramps *et al* (2002)
B9L/BCL9-2 (BCL9-like protein)	Binds *β*-catenin, increases transcription, induces EMT	Adachi *et al* (2004); Brembeck *et al* (2004)
Brg-1 (chromatin remodelling factor)	Binds *β*-catenin, increases transcription	Barker *et al* (2001)
c-Jun (phosphorylated)	Binds *β*-catenin and TCF/LEF1 in a JNK- and *β*-catenin-dependent manner, knockout decreases tumour multiplcity in *Apc*^*min*^ animals	Nateri *et al* (2005); Toualbi *et al* (2007)
c-Fos	Binds *β*-catenin, increases transcription	Toualbi *et al* (2007)
CARM1 (coactivator-associated arginine methyltransferase)	Binds *β*-catenin, increases transcription	Koh *et al* (2002)
CBP (CREB-binding protein)	Binds *β*-catenin	Takemaru and Moon (2000)
cdx1 and cdx2 (homeodomain transcription factors)	Decreases *β*-catenin Tyr phosphorylation, decreases transcription, induces E-cadherin adhesion	Guo *et al* (2004); Ezaki *et al* (2007)
Chibby (nuclear protein)	Binds *β*-catenin, inhibits transcription	Takemaru *et al* (2003)
CREB (cyclic AMP response element binding protein)	Binds *β*-catenin, induces expression of WISP-1 (Wnt-1-induced secreted protein 1)	Xu *et al* (2000)
cul4B (Cullin4B/E3-ubiquitin ligase)	Binds *β*-catenin and induces its proteolytic degradation	Tripathi *et al* (2007)
Duplin (axis duplication inhibitor)	Binds *β*-catenin in nucleus and inhibits transcription	Sakamoto *et al* (2000)
EBP50 (PDZ-containing protein)	Binds *β*-catenin, increases transcription	Shibata *et al* (2003)
emerin (type II inner nuclear membrane protein)	Binds *β*-catenin resulting in its cytoplasmic retention and decreased transcriptional activity	Markiewicz *et al* (2006)
ER*α* (estrogen receptor)	Binds *β*-catenin, increases transcription	Kouzmenko *et al* (2004)
ezh2 (enhancer of zeste homolog 2, polycomb group protein)	Binds *β*-catenin and ER*α*	Shi *et al* (2007)
FHL2 (four and a half of LIM-only protein 2, LIM coactivator)	Binds *β*-catenin, increases transcription	Wei *et al* (2003); Martin *et al* (2002)
FOXO (insulin- and oxidative stress signaling-induced transcription factor)	Binds *β*-catenin resulting in increased FOXO target gene transcription	Essers *et al* (2005)
FUS (fusion/translocated in liposarcoma, TLS)	Binds and increases *β*-catenin, regulates pre-mRNA splicing	Sato *et al* (2005)
GRIP1 (p160 coactivator of AR)	Binds *β*-catenin, augments AR activity	Li *et al* (2004)
Groucho/TLE (transcriptional repressor)	Binds *β*-catenin and is subsequently displaced from TCF/LEF1	Daniels and Weis (2005)
HIF1*α* (hypoxia inducible factor)	Binds *β*-catenin, competes with TCF/LEF1, induces survival and cellular adaptation to hypoxia	Kaidi *et al* (2007)
hARD1 (human arrest defective 1, acetyltransferase)	Binds and acetylates *β*-catenin, increases transcription	Lim *et al* (2006)
I-mfa (inhibitor of MyoD Family a)	Binds *β*-catenin, relieving I-mfa-mediated gene repression	Pan *et al* (2005)
ICAT (inhibitor of *β*-catenin and TCF-4)	Binds *β*-catenin, represses transcription	Tago *et al* (2000)
IKK*α* (I*κ*B kinase *α*)	Binds *β*-catenin, inhibits its ubiquitination, increases transcription	Lamberti *et al* (2001)
IKK*β* (I*κ*B kinase *β*)	Binds *β*-catenin, inhibits transcription	Lamberti *et al* (2001)
LRH-1 (orphan nuclear receptor)	Binds *β*-catenin, induces proliferation	Botrugno *et al* (2004)
LZTS2 (leucine zipper tumor suppressor 2)	Binds *β*-catenin, inhibits transcription	Thyssen *et al* (2006)
Mediator (MED12 subunit)	Binds *β*-catenin, increases transcription	Kim *et al* (2006)
Mitf (microphthalmia-associated transcription factor)	Binds *β*-catenin and competes with TCF/LEF1 to activate mitf target genes, important for melanocyte development	Schepsky *et al* (2006)
NF*κ*B, p50 subunit	Binds *β*-catenin, decreases NF*κ*B DNA binding, transactivation activity, regulates TNF*α*-induced CRP (C-reactive protein, acute-phase response protein) expression	Deng *et al* (2002); Sun *et al* (2005); Choi *et al* (2007)
Nurr1 (orphan nuclear receptor)	Binds *β*-catenin, increases transcription	Kitagawa *et al* (2007)
oct3/4	Binds *β*-catenin in ES cells, upregulates Nanog	Takao *et al* (2007)
p68 (DEAD box family of RNA helicases)	Binds *β*-catenin upon PDGF-induced Tyr phosphorylation of p68, increases transcription and EMT	Yang *et al* (2006)
p300	Binds and acetylates *β*-catenin, increases transcription	Sun *et al* (2000); Hecht *et al* (2000)
Parafibromin (component of polymerase-associated factor 1 (PAF1) complex)	Binds *β*-catenin, increases transcription, Pogypus dependent	Mosimann *et al* (2006)
Pin1 (prolyl isomerase)	Binds *β*-catenin, displaces it from APC, stabilises it and induces transcription, overexpressed in human tumours	Ryo *et al* (2001)
Pitx2 (bicoid-related transcription factor)	Induced by Wnt/Dvl/*β*-catenin, increases transcription	Kioussi *et al* (2002)
Pontin52 (nuclear protein)	Binds *β*-catenin	Bauer *et al* (1998)
PPAR*γ* (peroxisome proliferator-activated receptor)	Binds *β*-catenin, decreases membrane bound and cytoplasmic fraction	Sharma *et al* (2004); Liu *et al* (2004)
PRA1 (Prenylated Rab acceptor 1)	Binds *β*-catenin, inhibits transcription	Kim *et al* (2006)
prop1 (Prophet of Pit1, homeodomain factor)	Binds *β*-catenin, activates expression of lineage-determining transcription factor Pit1, represses the lineage-inhibiting transcription factor Hesx1 via TLE/Reptin/HDAC1 corepressor complexes	Olson *et al* (2006)
Pygopus	Complexes with *β*-catenin and TCF/LEF1 in a Legless-dependent manner	Kramps *et al* (2002); Thompson *et al* (2002)
RanBP3 (Ran binding protein 3)	Cofactor of chromosome region maintenance 1 (CRM1)-mediated nuclear export binds *β*-catenin in a RanGTP-stimulated manner, inhibits transcriptional activity	Hendriksen *et al* (2005)
RAR (retinoid acid receptor)	Binds *β*-catenin in retinoid-dependent manner, competes for binding with TCF/LEF1	Easwaran *et al* (1999)
Reptin52 (homologue of pontin52)	Binds *β*-catenin and Pontin52, inhibits transcription	Bauer *et al* (2000)
RXR*α* (retinoid X receptor)	Binds *β*-catenin, targets it for proteolytic degradation	Xiao *et al* (2003)
SHP-1 (protein-tyrosine phosphatase)	Binds *β*-catenin and inhibits transcription in intestinal epithelial cells	Duchesne *et al* (2003)
Smad1	Complexes with *β*-catenin and TCF/LEF1 resulting in increased myc expression	Hu and Rosenblum (2005)
Smad3	Binds *β*-catenin and TCF/LEF1	Labbe *et al* (2000); Jian *et al* (2006)
Smad4	Interacts with TCF/LEF1 (strong) and *β*-catenin (weak), coregulates TGF*β*/Wnt common target genes	Nishita *et al* (2000)
Smad7	Binds *β*-catenin, important for TGF*β*-induced apoptosis and targets *β*-catenin for proteolytic degradation	Edlund *et al* (2005); Han *et al* (2006)
Sox4	Binds and stabilises *β*-catenin and TCF/LEF1	Sinner *et al* (2007)
Sox9	Binds *β*-catenin and targets it for degradation	Akiyama *et al* (2004)
Sox17	Binds *β*-catenin and TCF/LEF1, targets its for proteolytic degradation	Sinner *et al* (2007)
TAK1 (MAPKKK) and NLK (Nemo-like kinase)	Interact with and phosphorylate TCF/LEF1 and *β*-catenin, inhibit DNA binding capacity and transcription	Ishitani *et al* (1999)
Teashirt (zinc finger protein)	Binds to armidillo (*Drosophila* homologue of *β*-catenin), activated by Wingless	Gallet *et al* (1998)
TCFs	Bind *β*-catenin	Molenaar *et al* (1996)
TIF2/GRIP1 (transcriptional intermediary factor-2/glucocorticoid receptor interacting protein-1)	Binds *β*-catenin and increases binding affinity to AR	Song and Gelmann (2005)
TOPO II*α* (DNA topoisomerase II*α*)	Binds *β*-catenin, increases transcription	Sato *et al* (2005); Huang *et al* (2007)
VDR (vitamin D receptor)	Binds *β*-catenin in a vitamin D-dependent manner, competes for binding with TCF/LEF1	Pálmer *et al* (2001)
XSox17*α*/*β* and Xsox3	Bind *β*-catenin and inhibit transcription	Zorn *et al* (1999)

EMT=epithelial-to-mesenchymal transition; FOXO=Forkhead box O; PDGF=platelet-derived growth factor; TGF*β*=transforming growth factor-*β*.

Proteins previously shown to directly bind to *β*-catenin in the nucleus are listed in alphabetical order together with a brief description and corresponding literature references. Please note that the list is admittedly incomplete as only direct binding partners have been included. Many other proteins have been excluded that do not directly bind to *β*-catenin but participate to its many complexes and may yet significantly affect its function.
